# Critical care, surgical management, xenotransfusion, and long-term monitoring in a maned wolf (*Chrysocyon brachyurus*) with severe traumatic injury

**DOI:** 10.1007/s11259-026-11345-7

**Published:** 2026-06-11

**Authors:** Delcio Almeida Magalhães, Isabella Abreu Castro, Beatriz Caroline Cabral Ibelli, Laura Castro Silva, Mariana Beatriz Rocha Sobrinho, Luca Neves Genari, Gustavo Ribeiro da Silva Tormem, Bruna dos Reis Gonçalves de Oliveira, Tamila Belchor de Araújo Alves, Diego Iwao Yamada, Márcio de Barros Bandarra

**Affiliations:** 1 Department of Veterinary Surgery and Animal Reproduction, School of Veterinary Medicine and Animal Science, District of Rubião Júnior, São Paulo State University (UNESP), Botucatu, SP Brazil; 2 Faculty of Veterinary Medicine and Animal Science, Federal University of Uberlândia, ³ Muriqui House, Ibiti Project, Conceição da Ibitipoca, Lima Duarte, Uberlândia, MG Brazil; 3 Muriqui House, Ibiti Project, Conceição da Ibitipoca, Lima Duarte, MG Brazil

**Keywords:** Wildlife emergency medicine, Perioperative stabilization, Blood transfusion strategies, Critical care in wildlife, Wild canids

## Abstract

This case report describes the advanced critical care, surgical management, and one-year outcome of a free-ranging adult male maned wolf (*Chrysocyon brachyurus*) presenting with severe polytrauma and concomitant renal dioctophymosis. The animal was rescued with a severely contaminated open tibial fracture associated with extensive soft tissue necrosis and active myiasis, rendering limb-sparing procedures unfeasible. A high pelvic limb amputation was therefore performed as a life-saving intervention. Diagnostic imaging and ultrasonography revealed extensive destruction of the right kidney caused by *Dioctophyma renale*, and right unilateral nephrectomy was subsequently undertaken following clinical stabilization. Thirteen adult nematodes were recovered from the renal capsule, along with one free parasite in the abdominal cavity. Due to severe anemia and hemodynamic instability, intraoperative xenotransfusion using packed red blood cells from a canine donor was performed after compatibility testing, resulting in cardiovascular stabilization without adverse reactions. Postoperative recovery was uneventful, and the animal demonstrated satisfactory functional adaptation to three-limbed ambulation under captive conditions. Serial clinical, hematological, biochemical, blood gas, and ultrasonographic evaluations over a one-year follow-up period revealed no evidence of renal dysfunction. This report provides novel clinical data on the use of xenotransfusion and complex surgical interventions in a maned wolf, contributing to the limited literature on advanced trauma management in wild canids.

## Background

Wild mammals play essential ecological roles in natural and human-modified landscapes, contributing to trophic regulation, seed dispersal, and ecosystem stability. Among Neotropical carnivores, canids are particularly relevant due to their wide distribution and ecological plasticity. The maned wolf (*Chrysocyon brachyurus*), the largest South American canid, is endemic to open habitats such as the Cerrado and grassland-dominated regions, with a historical range encompassing much of central South America (Queirolo et al. 2011). Its omnivorous diet allows the species to function both as a mesopredator and an effective seed disperser (Queirolo et al. 2011; Hammond 2011). Despite this adaptability, the species is currently classified as Near Threatened due to increasing anthropogenic pressures, including agricultural expansion, infrastructure development, and road networks, which have resulted in habitat fragmentation, restricted movement, and increased exposure to traumatic injuries (Rodriguez-Castro et al. 2022; Medrano-Vizcaíno et al. 2022). Although frequently recorded in agroecosystems, this reflects behavioral plasticity rather than true habitat preference, as maned wolves preferentially use unpaved roads and riparian corridors while avoiding areas of intense human activity (Pônzio et al. 2023).

Road-associated trauma in medium and large-bodied mammals commonly leads to severe orthopedic injuries, extensive soft tissue damage, hemorrhage, and systemic compromise, often requiring advanced critical care support and complex surgical management to achieve survival (Medrano-Vizcaíno et al. 2022; Rodriguez-Castro et al. 2022). In free-ranging maned wolves, traumatic injuries may occur concurrently with other conditions such as dioctophymosis caused by *Dioctophyma renale*, a parasitic disease acquired through ingestion of intermediate or paratenic hosts and capable of causing progressive renal parenchymal destruction (Hammond 2011). In domestic dogs, unilateral nephrectomy is considered the treatment of choice and is generally associated with favorable long-term outcomes when the contralateral kidney is functional, while limb amputation is a well-established life-saving procedure with satisfactory functional adaptation in most patients (Raske et al. 2015; Caye et al. 2024). However, in wild canids, and particularly in the maned wolf, data regarding postoperative survival, physiological compensation, and functional outcomes following major surgical interventions remain scarce.

Severe polytrauma associated with hemorrhagic compromise may necessitate blood transfusion as part of emergency stabilization. In zoological and wildlife medicine, transfusion practices are frequently extrapolated from domestic species due to the lack of species-specific blood banking systems, and xenotransfusion may represent a life-saving strategy when conspecific blood is unavailable (Petit and Charpentier 2022). Nevertheless, reports of xenotransfusion in free-ranging wild canids are rare, especially in maned wolves.

This case report describes the advanced critical care, surgical management, and use of xenotransfusion in a free-ranging maned wolf presenting with severe polytrauma and concomitant renal dioctophymosis, with a one-year postoperative follow-up.

## Case presentation

A free-ranging adult male maned wolf (*Chrysocyon brachyurus*), weighing 21 kg and presenting a lean body condition score (BCS 4/9), was captured from an industrial eucalyptus plantation in the Triângulo Mineiro region, Minas Gerais, Brazil. The animal was lethargic and presented with an extensive traumatic wound affecting the right pelvic limb. Chemical restraint was required to allow safe access, handling, and transport, using ketamine (5 mg/kg), dexmedetomidine (2 µg/kg), and midazolam (0.3 mg/kg), administered intramuscularly. Field assessment revealed a complete open fracture of the right tibia, and comfort immobilization was applied prior to hospital transport.

**Fig. 1:**
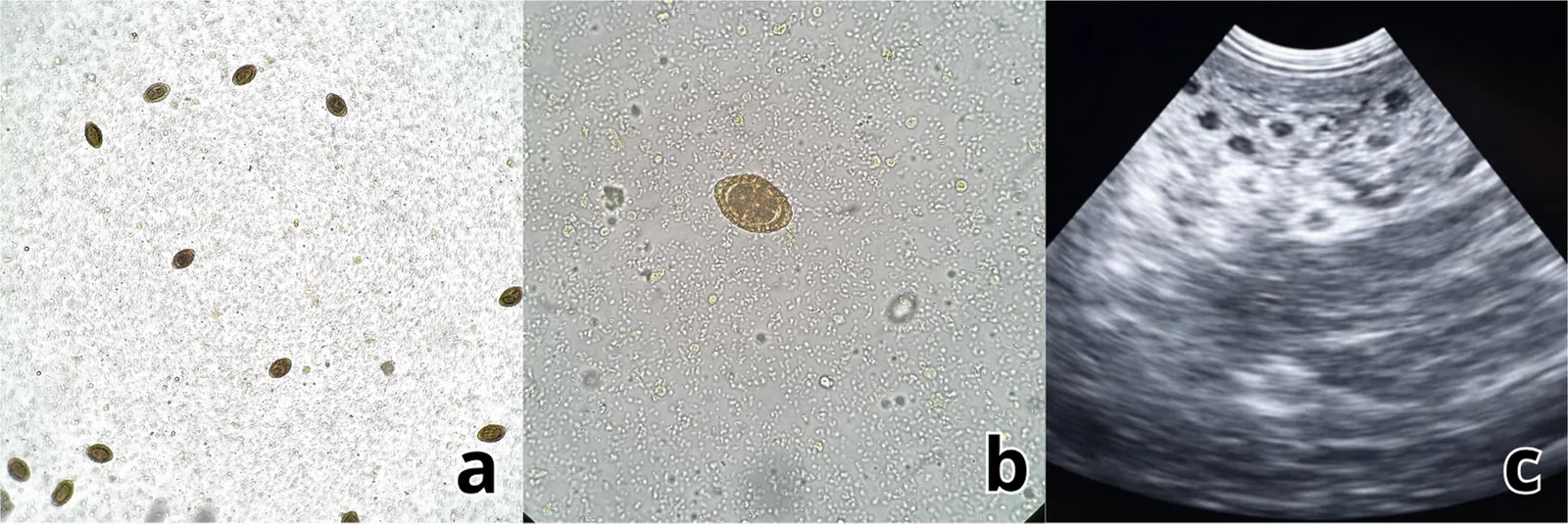
Diagnostic findings in a free-ranging adult male maned wolf (*Chrysocyon brachyurus*) with giant kidney worm infection: (**a**) and (**b**) *Dioctophyma renale* eggs in urine sediment (10× and 40×), and (**c**) ultrasonographic absence of the right kidney with loss of renal architecture consistent with severe parasitic destruction

On hospital admission, the orthopedic injury was classified as a severe open fracture with extensive bone necrosis and active myiasis involving the distal tibial diaphysis and bone marrow (Fig. 1**)**. A comprehensive diagnostic workup was conducted in accordance with the institutional triage protocol for wild canids, aiming to assess systemic involvement and guide therapeutic decision-making. This evaluation included a complete blood count, serum biochemistry panel, venous blood gas analysis, coagulation profile, urinalysis, urine protein-to-creatinine ratio (UPC) and molecular screening by polymerase chain reaction (PCR) for *Ehrlichia* spp., *Babesia* spp., *Anaplasma* spp., *Leishmania* spp., and *Hepatozoon* spp., all of which yielded negative results. Overall, laboratory findings were indicative of a severe systemic inflammatory response secondary to extensive traumatic injury and widespread soft tissue necrosis.

Hematological, biochemical, blood gas, coagulation, and urinary abnormalities were consistent with macrocytic normochromic anemia with schistocytes, inflammation and stress leukogram changes, hepatocellular injury or hypoxic insult, pre-renal azotemia, moderate metabolic acidosis with partial respiratory compensation, mild electrolyte imbalances, hyperfibrinogenemia with increased D-dimer concentrations, and marked proteinuria associated with renal injury (Tables 1, 2, 3, 4, 5 and 6). Urinalysis additionally revealed a high concentration of *Dioctophyma renale* eggs, confirming active environmental shedding of the parasite (Table 6**and** Fig. 1).

**Table 1:** Hematologic findings of a adult free-ranging maned wolf (*Chrysocyon brachyurus*) compared with reference values. Day 0: macrocytic normochromic anemia with schistocytes, neutrophilia, and platelet clumping. Day 4: severe macrocytic normochromic anemia with leukocytosis. Day 10: macrocytic hyperchromic anemia with schistocytes and persistent neutrophilia. 1 year: follow-up evaluation

Parameters	Day 0	Day 4	Day 10	1 year	Reference (Novais 2005)
RBC (×10^6/uL)	3.93	2.02	3.15	4.61	5.5–8.5
Hgb (g/dL)	11.1	5.6	8.9	12.6	12–18
PCV (%)	35	18	26.50	40	37–55
MCV (fL)	89.1	89.1	84.2	86.8	60–77
MCH (pg)	28.2	27.7	28.2	27.3	19.5–24.5
MCHC (g/dL)	31.7	31.1	33.5	31.5	30–36
Schistocytes	1+	0	1+	0	0
Codocytes	0	0	0	0	0
Polychromasia	0	2+	1+	0	0
Echinocytes	0	0	0	0	0
Anisocytosis	0	3+	2+	0	0
WBC (×10^3/uL)	14.0	18.2	17.0	8.9	8–17
Bands (%)	0	0	0	0	0–3
Bands (/µL)	0	0	0	0	-
Segmented (%)	93	90	96	76	60–77
Segmented (/µL)	13,020	16,380	16,320	6764	-
Eosinophils (%)	0	2	0	8	2–10
Eosinophils (/µL)	0	364	0	712	-
Basophils (%)	0	0	0	0	0–1
Basophils (/µL)	0	0	0	0	-
Monocytes (%)	2	6	3	1	3–10
Monocytes (/µL)	280	1092	510	89	-
Lymphocytes (%)	5	2	1	15	12–30
Lymphocytes (/µL)	700	364	170	1335	-
Platelets (x 10³/µL)	249	195	300	175	-
MPV (fL)	7.8	15.3	-	15.2	-
PDW (fL)	7.8	20.7	-	21.9	-
Plasma Protein	-	6.6	6.4	7	5.5–8.0
Platelet aggregation	Present	Absent	Intense	Absent	Absent
Plasma observations	-	-	Intense lipemia	-	Absent

*µL* microliter, *mg/dL* milligrams per deciliter, *fL* femtoliters, *pg* picogram, *g/dL* grams per deciliter

**Table 2:** Reticulocyte count of a adult free-ranging maned wolf (*Chrysocyon brachyurus*) compared with reference values indicating moderate regenerative anemia (60,000–200,000/µL)

Parameters	Day 0	Day 8	Reference
Total Erythrocytes (×10^6/µL)	2.02	2.49	-
PCV (%)	18	20.5	-
Corrected Reticulocyte Percentage (%)	1.3	4	-
Absolute Reticulocyte Count (/µL)	60,600	219,120	60,000–200,000: modarate anemia (Domestic dog reference)

*µL* microliter

**Table 3:** Biochemical profile of a adult free-ranging maned wolf (*Chrysocyon brachyurus*) compared with reference values. Day 0: hypoalbuminemia with increased ALP, ALT, and urea. Day 4: persistent hypoalbuminemia with rising ALP, ALT, GGT, and urea. Day 11: decreased albumin and creatinine with hypercalcemia. 1 year: follow-up evaluation

Parameters	Day 0	Day 4	Day 11	1 year	Reference (Novais 2005; Silva 2007)
Albumin (g/dL)	2.26	1.88	1.53	2.65	2.86–3.94
Alkaline Phosphatase (U/L)	112	190	-	40	10–96
ALT (U/L)	377	722	-	90	7–92
Creatinine (mg/dL)	1.49	1.47	0.93	0.54	1.09–1.71
GGT (U/L)	3.1	4.1	-	3.6	2.1–3.5
Urea (mg/dL)	63.7	138.3	59.2	65.5	10–60
Phosphorus (mg/dL)	-	-	5.16	5.57	5.0–5.8
Calcium (mg/dL)	-	-	7.58	8.6	2.2–2.4

*U/L* unit per liter, *mg/dL* milligrams per deciliter, *g/dL* grams per decilitre

**Table 4:** Venous blood gas analysis of a adult free-ranging maned wolf (*Chrysocyon brachyurus*) compared with domestic dog reference values. Day 0: acidemia with hyponatremia and ionized hypocalcemia. Day 4: persistent electrolyte imbalance with increased anion gap. Day 6: persistent hypocalcemia and metabolic acidosis. 1 year: follow-up evaluation

Parameters	Day 0	Day 4	Day 6	1 year	Reference
Sodium (mmol/L)	130.2	139.1	140.0	145.6	140–155(Domestic dog reference)
Potassium (mmol/L)	5.79	4.78	4.70	4.92	3.5–5.8 (Domestic dog reference)
Chloride (mmol/L)	108.6	116.2	119.4	115.3	100–120 (Domestic dog reference)
Ionized Calcium (mmol/L)	1.19	1.21	1.22	1.24	1.3–1.5 (Domestic dog reference)
Glucose (mg/dL)	106	81	66	115	-
Lactate (mmol/L)	2.0	3.6	1.5	1.4	-
Anion Gap (mmol/L)	12.0	7.9	7.0	10.9	12–25 (Domestic dog reference)
pH (T)	7.22	7.37	7.39	7.334	7.351–7.443 (Domestic dog reference)
PCO2 (T) (mmHg)	37.8	33.9	29.5	45.2	33.6–41.2 (Domestic dog reference)
PO2 (T) (mmHg)	65.1	42.6	39.2	42.6	47.9–56.3 (Domestic dog reference)
HCO3 (mmol/L)	15.5	19.8	18.4	24.3	21–25 (Domestic dog reference)
BE-b (mmol/L)	10.0	4.3	5.4	1.1	-

*mmol/L* millimoles per liter, *mmHg* millimeters of mercury, *mg/dL* milligrams per deciliter

**Table 5:** Coagulation profile of a adult free-ranging maned wolf (*Chrysocyon brachyurus*) compared with reference values. Day 0: prolonged PT and aPTT with hyperfibrinogenemia. Day 8: persistent abnormalities. Day 32: persistent PT and aPTT prolongation with normalized fibrinogen. 1 year: follow-up evaluation

Parameters	Day 0	Day 8	Day 32	Reference
Prothrombin Time - PT (s)	9.4	9.0	9.8	5.7–8.0 (Domestic dog reference)
Activated Partial Thromboplastin Time - aPTT (s)	18.4	21.1	22.5	10.0–14.3 (Domestic dog reference)
Fibrinogen (mg/dL)	768.1	452.9	247.7	130–310 (Domestic dog reference)
D-Dimer (ng/mL)	429	227	20.0	23–650 (Domestic dog reference)

*mg/dL* milligrams per deciliter, *ng/mL* nanograms per milliliter, *s* seconds

**Table 6:** Urinalysis and UPC of a adult free-ranging maned wolf (*Chrysocyon brachyurus*) compared with domestic dog reference values. Day 0: dark yellow urine with marked bilirubinuria and presence of *Dioctophyma renale* eggs.1 year: follow-up evaluation

Parameters	Day 0	1 year	Reference
Color	Dark yellow	Gold yellow	Yellow (Domestic dog reference)
Aspect	Turbid	Semi-turbid	Turbid (Domestic dog reference)
Density	1.040	1.040	1,030 (Domestic dog reference)
pH	7.0	7.0	7,5 (Domestic dog reference)
Protein	1+	Traits	1+ (Domestic dog reference)
Glucose	Negative	Negative	Negative (Domestic dog reference)
Ketone	Negative	1+	-
Occult blood	4+	2+	-
Urobilinogen	2+	Abnormal	
Bilirubin	3+	2+	Negative (Domestic dog reference)
Bile salts	Negative	Negative	-
Squamous epithelial cells (average per field)	Absent	Rare	-
Transitional epithelial cells (average per field)	Rare	0–6 p	-
Renal pelvis epithelial cells (average per field)	Absent	Absent	-
Renal tubular epithelial cells (average per field)	Absent	Absent	-
Pyocytes (average per field)	5–20	< 5	-
Red blood cells (average per field)	5–20	< 5	
Bacteria	4+	1+	-
Casts	Absent	Granular: 1+	
Crystals	Bilirubin 1+	Absent	
Observations	Presence of *Dioctophyma renale* eggs	-	-
Urinary Protein (mg/dL)	101	19	-
Urinary Creatinine (mg/dL)	233	169.9	
UPC Ratio	0.43	0.11	< 0.5 (Domestic dog reference)

*mg/dL* milligrams per deciliter

**Fig. 2:**
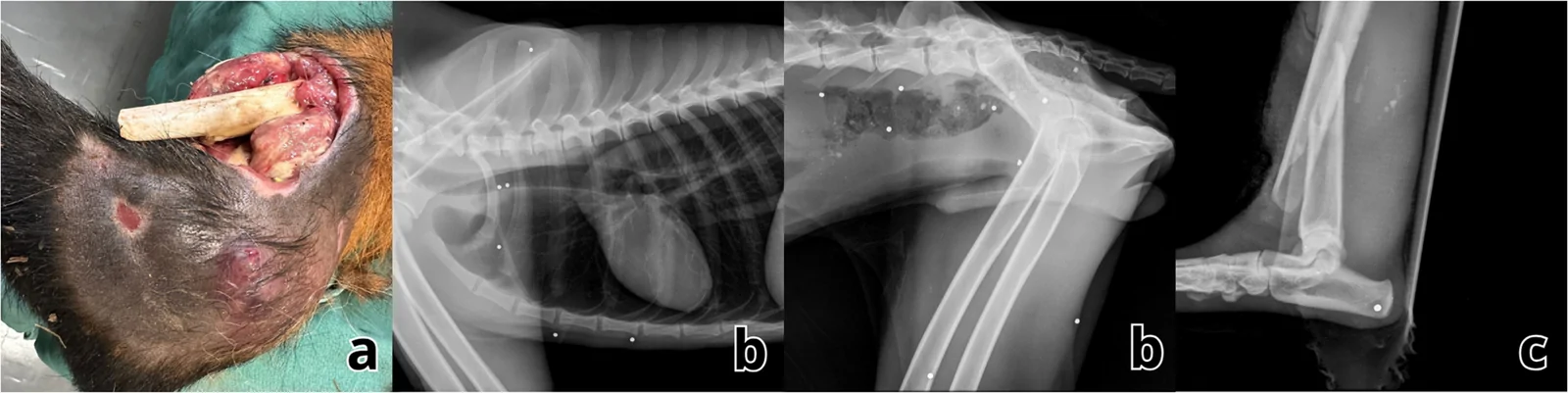
Orthopedic and radiographic findings in an adult male maned wolf (*Chrysocyon brachyurus*) showing (**a**) open distal tibial diaphyseal fracture with bone necrosis, (**b**) multiple ballistic projectiles in soft tissues, abdomen, humerus, and right tibia, and (**c**) complete distal tibial diaphyseal fracture

A global Focused Assessment with Sonography for Trauma (FAST) examination was performed as part of the initial stabilization protocol. Ultrasonography revealed absence of the right kidney from its expected anatomical location, loss of renal contours, reactive mesentery, and an elongated tubular structure adjacent to the proximal duodenum and caudate liver lobe, findings compatible with severe renal destruction caused by *Dioctophyma renale* (Fig. 1). Thoracic and abdominal radiographs demonstrated discrete microcardia consistent with hypovolemia and/or dehydration, as well as multiple ballistic projectiles distributed within soft tissues, the abdominal cavity, humerus, and right tibia, compatible with penetrating intentional trauma (Fig. 2).

Initial stabilization focused on analgesia, infection control, and hemodynamic support. Multimodal analgesia was instituted with tramadol (4 mg/kg) and metamizole/dipyrone (25 mg/kg), antimicrobial therapy with ceftriaxone (30 mg/kg) and metronidazole (20 mg/kg), and intravenous fluid therapy with lactated Ringer’s solution (5 mL/kg/h). After an eight-hour fasting period and clinical stabilization, surgical intervention was scheduled.

Pelvic limb amputation was performed under partial intravenous anesthesia combined with inhalant anesthesia and multimodal analgesia. Premedication consisted of dexmedetomidine (0.7 µg/kg), midazolam (0.3 mg/kg), ketamine (3.5 mg/kg), and methadone (0.2 mg/kg). Ultrasound-guided regional anesthesia was performed using the GIN-TONIC block, Greater Ischiatic Notch - Targeted Obstruction of Nerve Conduction, an interfascial regional anesthesia technique described in veterinary medicine to provide pelvic limb analgesia by blocking the sciatic nerve, with 0.25% bupivacaine at 3 mg/kg. The patient was intubated with a 7.5 mm internal diameter orotracheal tube, and anesthesia was maintained with isoflurane in oxygen. Intraoperative management included lactated Ringer’s solution at 3 mL/kg/h and continuous rate infusions of ketamine at 0.6 mg/kg/h, remifentanil at 10 µg/kg/h, and propofol at 0.2 mg/kg/h. Continuous multiparametric monitoring was performed, including pulse oximetry, electrocardiography, non-invasive blood pressure measurement using Doppler, capnography, temperature monitoring, and assessment of corneal, palpebral, and withdrawal reflexes, with parameters recorded every five minutes.

Marked anesthetic instability was observed during the procedure, characterized by hypotension, with systolic arterial pressure of 60 mmHg and diastolic arterial pressure of 40 mmHg. A crystalloid bolus of lactated Ringer’s solution at 10 mL/kg was administered over 30 min, followed by initiation of norepinephrine infusion at 0.2 µg/kg/min, and after two hours of surgery, dobutamine was added at 5 µg/kg/min. Hypoventilation was detected with an end-tidal carbon dioxide concentration of 70 mmHg, requiring institution of volume-controlled mechanical ventilation with a tidal volume of 197 mL, positive end-expiratory pressure of 4 cmH₂O, peak inspiratory pressure of 6 cmH₂O, and fraction of inspired oxygen of 60%, which was maintained throughout the procedure. Bradycardia of 32 beats per minute was treated with atropine at 0.022 mg/kg, administered twice at a 10-minute interval.

A high pelvic limb amputation via coxofemoral disarticulation was performed in accordance with standard surgical principles. A marked hemorrhagic episode occurred during joint disarticulation, contributing to transient intraoperative hypotension; however, no additional intraoperative complications were observed. Postoperatively, analgesic and antimicrobial therapy were maintained. Owing to the patient’s good tolerance and handling permissiveness, parenteral administration was elected for ongoing therapeutic management.

Seventy-two hours after surgery, hematological reassessment revealed progression to severe anemia, with a hematocrit of 18%. Despite the absence of overt clinical instability, repeat FAST identified a small volume of free abdominal fluid in the hepato-renal umbilical (HRU) view. Immediate surgical intervention was not performed, as the patient remained hemodynamically stable and was maintained under close clinical and laboratory monitoring. This period also allowed for preoperative planning, including evaluation and selection of potential canine donors in anticipation of a possible xenotransfusion, while supportive therapy was continued.

Five days after the initial procedure, exploratory laparotomy and right unilateral nephrectomy were performed under general anesthesia. Premedication consisted of dexmedetomidine (0.7 µg/kg), midazolam (0.28 mg/kg), ketamine (3.5 mg/kg), and methadone (0.2 mg/kg). Anesthesia was induced with propofol to effect and maintained with isoflurane in oxygen. Crystalloid fluid therapy was administered at approximately 3 mL/kg/h, and analgesia was supplemented with a continuous fentanyl infusion (5 µg/kg/h). Persistent intraoperative hypotension necessitated vasopressor support with norepinephrine, titrated at 0.2 µg/kg/min.

Given the severity of anemia and possible ongoing hemodynamic instability, an intraoperative packed red blood cell transfusion was instituted using a xenotransfusion protocol with a healthy adult 48-kg Rottweiler canine donor. Major and minor crossmatching, as well as saline agglutination testing, were performed prior to transfusion and revealed no evidence of incompatibility. Transfusion was initiated at a conservative rate of 1 mL/kg/h and gradually increased to a maximum of 10 mL/kg/h under continuous anesthetic and hemodynamic monitoring. Cardiovascular and respiratory parameters, including heart rate, invasive arterial blood pressure, capnography, pulse oximetry, and body temperature, were closely monitored throughout the procedure, along with careful assessment for clinical signs of acute transfusion reactions. A total volume of 220 mL of packed red blood cells was administered without evidence of acute transfusion-related adverse events, including hemolysis, hypersensitivity reactions, or cardiorespiratory compromise.

**Fig. 3:**
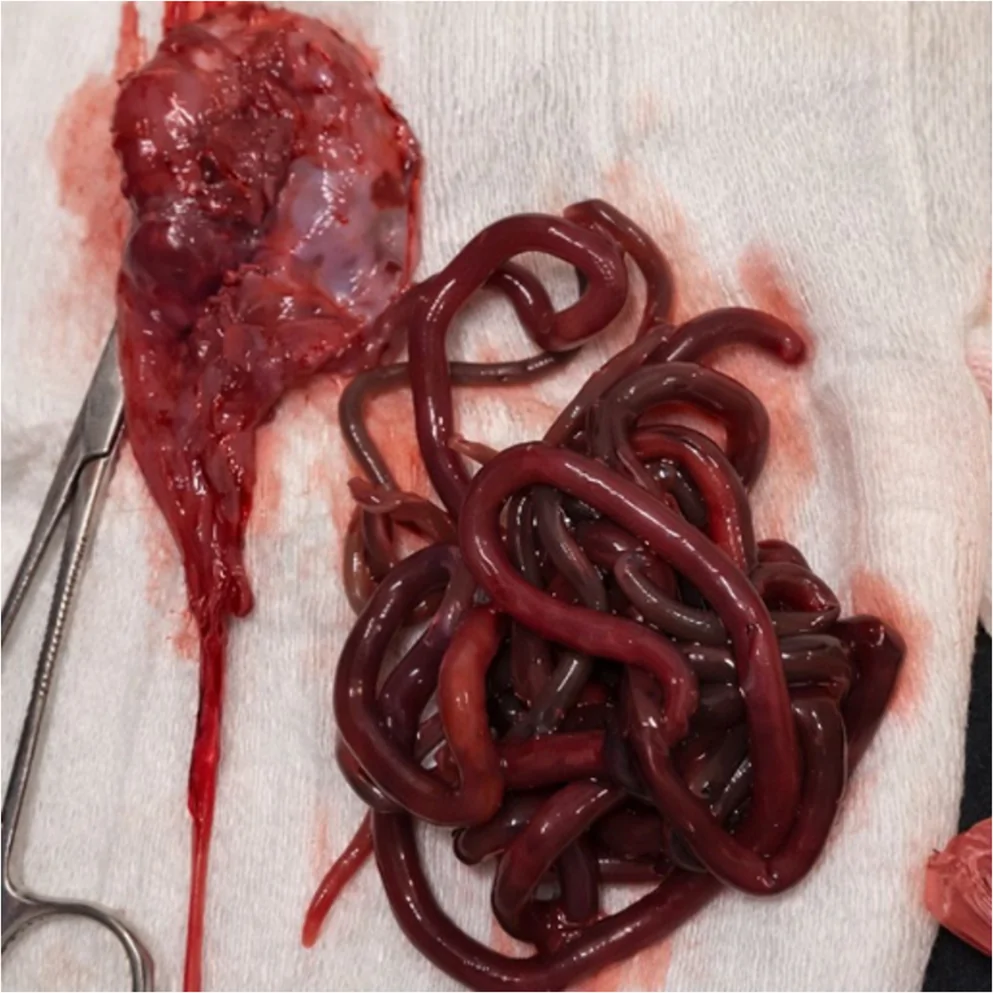
Right kidney of a free-ranging adult male maned wolf (*Chrysocyon brachyurus*) after unilateral nephrectomy showing multiple adult *Dioctophyma renale*

Right unilateral nephrectomy was performed via ventral midline approach. Thirteen *Dioctophyma renale* specimens were identified within the renal capsule, and one adult nematode was found free within the abdominal cavity (Fig. 3). Routine abdominal lavage with sterile NaCl 0,9% 200 mL/kg and closure were performed without complications.

Postoperative management included cefpodoxime (10 mg/kg q24h for 10 days), meloxicam (0.1 mg/kg q24h for 3 days), tramadol hydrochloride (4 mg/kg q12h for 4 days), and metamizole/dipyrone (25 mg/kg q12h for 5 days). All medications were administered orally, incorporated into a meat and fruit-based diet, with direct confirmation of complete ingestion. Serial laboratory testing and ultrasonographic evaluations confirmed progressive clinical improvement.

**Fig. 4:**
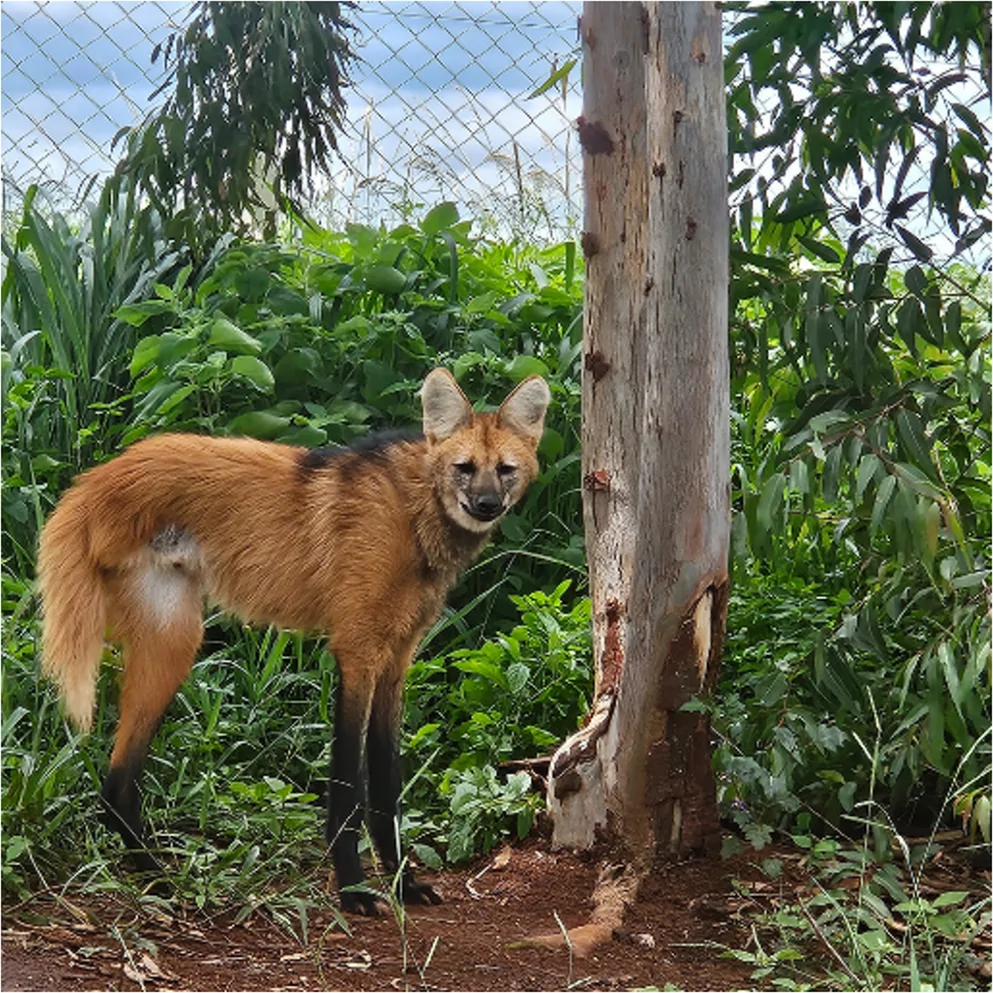
Adult male maned wolf (*Chrysocyon brachyurus*) one year after pelvic limb amputation demonstrating successful tripodal locomotion and ideal body condition

At 30, 60, 90, and 150 days and one year postoperatively, the animal, maintained in captivity due to its clinical condition, remained clinically stable, with unremarkable hematological, biochemical, coagulation, blood gas, and ultrasonographic findings. No evidence of renal dysfunction was observed, and the maned wolf demonstrated satisfactory long-term functional adaptation to three-limbed ambulation, appropriate behavior, and maintenance of good body condition score (Fig. 4).

### Discussion and conclusion

Severe open fractures associated with extensive contamination, bone necrosis, and irreversible loss of viable soft tissue are consistently linked to poor prognosis and limited feasibility of limb-sparing procedures. In such circumstances, amputation is widely recognized as a life-saving intervention once the biological envelope is compromised beyond recovery (Vinayak 2024). In free-ranging carnivores, however, surgical decision-making must extend beyond technical feasibility and include the anticipated effects of trauma, hemorrhage, and systemic instability on survival, particularly when advanced critical care resources are required and long-term post-release monitoring is limited (Hammond 2011; Vetter Hiebert et al. 2024).

Although limb amputation may result in satisfactory functional outcomes in domestic dogs, extrapolation to wild canids requires caution. Objective gait analyses in dogs demonstrate substantial redistribution of body weight following high pelvic limb amputation, with increased loading of contralateral limbs and forelimbs (Túlio Filho et al. 2024). While the maned wolf described herein showed rapid postoperative adaptation and stable ambulation under captive conditions, functional compensation observed in controlled environments does not reliably predict performance in natural settings that demand efficient locomotion for foraging, territorial defense, predator avoidance, and road crossing. Consequently, despite clinical stabilization and surgical success, reintroduction was considered contraindicated in the absence of continuous post-release monitoring (Hammond 2011; Vetter Hiebert et al. 2024).

Dioctophymosis remains an important and frequently underdiagnosed condition in free-ranging carnivores, particularly in regions where domestic and wild transmission cycles overlap. In maned wolves, most diagnoses are historically obtained at necropsy, and although antemortem detection has increased in recent years, relatively few published reports describe therapeutic intervention with documented outcomes (Duarte et al. 2013). When clinically identified, renal parenchymal destruction is often advanced, as observed in the present case, reflecting the progressive nature of the infection and delayed detection. Molecular studies demonstrate high genetic diversity of *Dioctophyma renale* and bidirectional transmission between domestic and wild carnivores, particularly in fragmented landscapes and riparian environments (Arce et al. 2025). In the Brazilian Cerrado, increasing habitat modification, forestry expansion, and urbanization have intensified interactions between domestic dogs and wildlife, facilitating parasite circulation at the domestic-wildlife interface (Assis-Silva et al. 2025). These ecological dynamics likely contributed to the infection observed in this case. While isolated pharmacological approaches have been reported, nephrectomy remains the recommended treatment for advanced renal involvement (Mesquita et al. 2014; Oliveira et al. 2021).

A central and clinically relevant aspect of this case was the requirement for advanced hemodynamic stabilization in the context of severe polytrauma, hemorrhage, and systemic inflammatory response, culminating in the use of xenotransfusion as a life-saving strategy prior to definitive abdominal surgery. In zoological and wildlife medicine, blood transfusion practices are frequently extrapolated from domestic species due to the lack of species-specific blood banking systems, and xenotransfusion may represent the only feasible option in emergency scenarios involving free-ranging animals (Petit and Charpentier 2022). Although immunological risks are inherent to xenotransfusion, previous reports in wild canids, including the Andean fox (*Lycalopex culpaeus*) (Díaz et al. 2020) and the island fox (*Urocyon littoralis*) (Martony et al. 2016), have demonstrated short-term clinical success with absence of immediate transfusion reactions.

In the present case, compatibility testing revealed no evidence of incompatibility, and xenotransfusion resulted in effective cardiovascular stabilization without transfusion-related adverse events. This intervention was pivotal in restoring hemodynamic stability and enabling safe progression to definitive surgical management. These findings support the cautious use of xenotransfusion as an emergency stabilization measure in critically ill wild canids when conspecific blood is unavailable, provided that appropriate compatibility testing and intensive monitoring are performed (Petit and Charpentier 2022).

In conclusion, this report demonstrates that advanced critical care stabilization, including xenotransfusion, combined with high pelvic limb amputation and unilateral nephrectomy can result in favorable short and mid-term clinical outcomes in a maned wolf when performed under carefully controlled conditions. Nevertheless, the lack of species-specific reference data limits prognostic accuracy, and clinical success in individual cases should not be generalized. Given the functional, ecological, and potential long-term renal implications of such extensive interventions, management decisions must be individualized and supported by rigorous postoperative monitoring. This case contributes novel clinical evidence regarding xenotransfusion in a free-ranging maned wolf and highlights the need for additional long-term, multicenter studies to better define outcomes and critical care strategies in surgically treated wild canids.

## Data Availability

No datasets were generated or analysed during the current study.
